# Lower cognitive function attenuates the convergence between self‐ratings and observer ratings of depressive symptoms in late‐life cognitive impairment

**DOI:** 10.1002/brb3.2898

**Published:** 2023-02-08

**Authors:** Seyul Kwak, Hairin Kim, Dae Jong Oh, Yeong‐Ju Jeon, Da Young Oh, Su Mi Park, Jun‐Young Lee

**Affiliations:** ^1^ Department of Psychology Pusan National University Busan Republic of Korea; ^2^ Department of Psychiatry Seoul Metropolitan Government‐Seoul National University Boramae Medical Center Seoul Republic of Korea; ^3^ Department of Counseling Psychology Hannam University Daejeon Republic of Korea

**Keywords:** Alzheimer's disease, cognitive impairment, dementia, depression, late‐life, self‐report, validity

## Abstract

**Objectives:**

Assessment of depressive symptoms in older adults is challenging especially in the presence of risks in cognitive impairment. We aimed to examine whether the convergence between two measures of depressive symptoms (self‐report and observer ratings) is affected by varying levels of cognitive function in older adults.

**Methods:**

Self‐reported scale of depression, informant‐based rating of affective symptoms, and global cognitive function were assessed in 2533 older adults with no impairment, mild cognitive impairment, and Alzheimer's disease. The strength of rank‐order correlation between the Geriatric Depression Scale (GDS) and behavioral ratings of the Neuropsychiatric Inventory (NPI) was examined as the metric of convergent validity.

**Results:**

The results showed that the strength of convergence between the two measurements gradually decreased as a function of lowered cognitive function. Overall tendency showed that diagnoses of cognitive impairment and lower levels of cognitive function were associated with lower correspondence between the two depression measurements. The loss of convergent validity is especially evident in the behavioral symptom of apathy.

**Conclusions:**

Utilizing self‐report scales of depression in older adults requires a cautious approach even with minimal or mild levels of cognitive impairment.

## Graphical abstract

The results showed that the strength of convergence between the two measurements gradually decreased as a function of lowered cognitive function. Utilizing self‐report scales of depression in older adults requires a cautious approach even with minimal or mild levels of cognitive impairment.

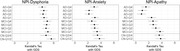

## 1 INTRODUCTION

Depression is a highly prevalent condition that disturbs an individual with emotional distress and daily functioning. Depressive symptoms in the elderly population often co‐occur with the progression of late‐life cognitive impairment, especially in patients with Alzheimer's disease (AD) or mild cognitive impairment (MCI) (Ismail et al., 2017; Van Der Mussele et al., 2013). Previous studies suggest that increased neurovascular risk or dementia pathology precipitates the onset of depression, while chronic depression can conversely accelerate late‐life cognitive decline (Brodaty & Connors, 2020; Rutherford et al., 2017; Ruthirakuhan et al., 2019). Valid assessment of depressive symptoms in older adults is a challenging issue, especially in the presence of risks in cognitive impairment.

The Geriatric Depression Scale (GDS) is a commonly used self‐rating scale developed to screen depression in older adults (Yesavage et al., 1982). With the advantage of an easy administration format (i.e., yes or no format with abbreviated items), the instrument has been widely used in evaluating depressive symptoms of older adults (Balsamo et al., 2018). While some studies showed that depression scales including the Center for Epidemiologic Studies—Depression Scale (CES‐D) were comparable in diagnostic utility, other studies suggested GDS as the most efficient screening scale under specific neurological conditions (Lyness et al., 1997; Williams et al., 2012). The utility of GDS more stood out considering the shorter administration time and psychometric properties when compared with other various scales including Beck Depression Inventory‐II (BDI‐II) or Patient Health Questionnaire‐9 (PHQ‐9). The GDS and other forms of self‐reported measures of depression symptom scales have shown evidence of reliability and validity in administering patients with cognitive impairment; the applicability remains questioned in how the measurement integrity generalizes to the wide range of the population.

The prerequisite of accurate measurement of depressive symptoms depends on the capacity to identify and conceptualize the item contents. For respondents, however, differentiating discrete emotional experiences and recollecting recent autobiographical memory requires significant abilities of abstraction and memory retrieval. Self‐descriptive statements require an individual to attribute specific behaviors and attitudes. In a population with a cognitive deficit, it may be limited in their accuracy to elaborate on one's depressive symptomatology (Emerson et al., 2013; Verhey et al., 1993).

Previous studies have aimed to examine whether the self‐report scales are applicable to older adults with risks of cognitive impairment (Bédard et al., 2003; Conradsson et al., 2013; Ott & Fogel, 1992). While some evidence suggests an acceptable validity of using the self‐report scale in the demented population (Lach et al., 2010), other approaches have shown opposing evidence that shows moderate convergence between self‐report and clinician rating (i.e., Cornell Scale for Depression in Dementia) (Burke et al., 1989; Debruyne et al., 2009). It remains elusive whether the self‐report scale validly converges with externally observed behavioral information across varying ranges of cognitive functioning. Considering the spectral dimensionality from cognitively normal (CN) to dementing population, the convergent validity of depression instruments may show a gradual difference as a function of cognitive function.

Another issue in the assessment of depression in later life is the complications in subsymptoms of depression. While multiple depressive symptoms, including dysphoria, apathy, and anxiety, typically co‐occur as a syndrome, their behavioral manifestation may have differential correspondence to the self‐report scale. It is possible that correspondence to self‐report scales may dissociate from one another especially in cognitively impaired populations due to different sources of etiology (Brodaty & Connors, 2020; Levy et al., 1998).

Among varying dementia types and neuropathological features, AD still constitutes the majority of dementia patients and the MCI population (Dugger et al., 2015). One of the challenges in the clinical assessment is to accurately identify the presence of depressive symptoms under compromised cognitive ability. While apathy, indifference, and lack of emotionality characterize the depressive symptoms of AD, dysphoria and feelings of worthlessness characterize the typical depressive syndrome, which forms the bases of differentiating diagnoses (Brodaty & Connors, 2020). A formal evaluation of validity is needed in instruments used for assessing affective disturbances of dementing population

The current study aimed to examine the strength of convergence between self‐reported depression symptoms (GDS) and behaviorally rated symptoms of depression. It is hypothesized that the presence of cognitive impairment or lower global cognitive functioning is associated with lower convergence between self‐report and informant rating.

## 2 METHODS

### 2.1 Participants

The older adults with or without cognitive impairment were retrospectively recruited from SMG‐SNU Boramae Medical Center for Dementia from January 2012 to December 2020. The participants underwent both neuropsychological assessment and structured clinical interviews. This study was conducted under the Declaration of Helsinki, and the protocol was approved by the Institutional Review Board of SMG‐SNU Boramae Medical Center (IRB No. 10‐2020‐295). The current study included older adults without cognitive impairment or diagnosed with cognitive impairment (MCI and, AD dementia). The clinical diagnosis of MCI and AD dementia was based on the core clinical criteria of National Institute on Aging‐Alzheimer's Association workgroups (NIA‐AA) guidelines (Albert et al., 2011; McKhann et al., 2011). Older adults without diagnoses of any cognitive impairment were considered CN. The presence of an objective cognitive decline and cognitive impairment in a specific cognitive domain was determined by demographics‐adjusted norms of age, education, and sex (Lee et al., 2004). The AD group shows disturbances of independent daily activities, whereas the MCI group showed overall intact functioning. Individuals within a normal range of cognitive functions were considered CN.

Subjects suspected or diagnosed with dementia types other than AD were not included in the analysis, including vascular dementia, Lewy body dementia, frontotemporal lobe dementia, and vascular dementia. In addition, those identified or suspected with significant neurological or psychiatric conditions including stroke, traumatic brain injury, meningioma, hemorrhage, normal pressure hydrocephalus, delirium, intellectual disabilities, and psychotic disorders were also excluded. We confined our predictive analysis within the dementia staging of “moderate” impairment (Clinical Dementia Rating sum of box score ≤15.5) (O'Bryant, 2008). Finally, a total of 2533 older adults who met the screening criterion were analyzed (Table 1).

**TABLE 1:** Descriptive characteristics across the clinical diagnosis of cognitive impairment and quartiles subgroups of cognitive function

Cognitive function groups	*n*	Age	Education	MMSE	CERAD‐K	GDS	NPI‐Dysphoria	NPI‐Anxiety	NPI‐Apathy
CN‐Q1/2	154	68.18 (7.77)	12.08 (4.13)	27.82 (1.52)	77.92 (5.41)	4.32 (3.77)	0.81 (0.94)	0.46 (0.79)	0.36 (0.72)
CN‐Q3/4	145	74.51 (6.44)	7.55 (4.67)	25.68 (2.92)	63.03 (5.57)	4.87 (3.85)	0.88 (0.90)	0.61 (0.84)	0.34 (0.71)
MCI‐Q1	257	70.56 (7.03)	10.37 (4.18)	26.19 (2.28)	68.33 (4.52)	5.98 (4.15)	0.99 (0.94)	0.56 (0.88)	0.46 (0.74)
MCI‐Q2	263	72.60 (7.54)	9.06 (4.42)	24.39 (2.79)	58.84 (2.04)	6.00 (4.17)	1.00 (0.96)	0.59 (0.89)	0.53 (0.83)
MCI‐Q3	272	74.36 (6.6)	7.42 (4.35)	23.19 (3.01)	51.86 (2.25)	6.45 (3.99)	1.05 (0.94)	0.62 (0.87)	0.56 (0.84)
MCI‐Q4	233	77.97 (6.68)	4.83 (4.65)	19.59 (3.65)	40.42 (5.60)	7.11 (4.25)	1.06 (0.93)	0.59 (0.86)	0.64 (0.90)
AD‐Q1	309	76.65 (7.21)	9.16 (4.83)	21.53 (3.36)	52.32 (5.94)	5.85 (4.24)	0.93 (0.91)	0.62 (0.93)	0.76 (0.91)
AD‐Q2	311	78.78 (6.99)	6.96 (4.81)	18.23 (3.46)	40.86 (2.54)	6.61 (4.29)	1.06 (0.91)	0.66 (0.90)	0.82 (0.93)
AD‐Q3	318	80.91 (6.52)	6.12 (4.82)	15.61 (3.37)	31.95 (2.59)	7.09 (3.98)	1.03 (0.89)	0.68 (0.87)	1.04 (0.97)
AD‐Q4	271	80.63 (6.57)	4.05 (4.64)	11.48 (3.69)	19.93 (6.38)	7.46 (4.10)	1.09 (0.99)	0.87 (0.98)	1.14 (1.02)
Total	2533	76.07 (7.93)	7.57 (5.08)	20.70 (5.73)	48.22 (16.53)	6.33 (4.19)	1.00 (0.93)	0.64 (0.89)	0.71 (0.92)

*Note*: Values indicate mean and standard deviation (parenthesis).

Abbreviations: AD, Alzheimer's disease; CERAD‐K, global cognitive function; CN, cognitively normal; GDS, Geriatric Depression Scale (Self‐report); MCI, mild cognitive impairment; NPI, Neuropsychiatric Inventory (Observer rating); Q1∼4, quartile splits within each clinical diagnosis (lower to higher score).

### 2.2 Global cognitive function

All participants received the Korean version of the Consortium to Establish a Registry for Alzheimer's Disease neuropsychological battery (CERAD‐K) (Lee et al., 2002). The battery measures multiple domains of cognitive function and facilitates the diagnosis of MCI and dementia. The battery contains the following subtests as constituents of the total score: Verbal fluency (the number of correct animal words), Boston Naming Test, Word List Recall (immediate, delayed), Word List Recognition (subtraction of the number of false positives from the number of true positives), and Constructional Praxis (copy).

The CERAD‐K total score was calculated as the sum of the raw scores subdomains as previously described (Chandler et al., 2005). The total score of the CERAD‐K has shown utility to detect and predict AD dementia with high diagnostic accuracy, which describes global cognitive function (Wolfsgruber et al., 2014). The total scores ranged from 3 to 98 across participants. The each clinical diagnosis (MCI and AD) were divided into subgroups based on the quartile boundaries (i.e., 25th, 50th, and 75th percentile). The CN group was divided into median‐split subgroups to comprise a comparable sample size. The Mini‐Mental Status Examination (MMSE) score was provided for descriptive purposes. Either version of MMSE‐KC (Korean version of the consortium) or MMSE‐DS (Dementia Screen) was utilized, which showed an interchangeably strong correlation (*r* = .955) (Tae et al., 2010).

### 2.3 Self‐reported depressive symptoms

The self‐reported depressive symptoms were assessed with the short‐form Korean version of the GDS (Bae & Cho, 2004). Questions from the original GDS that had the highest correlation with depressive symptoms in validation studies were selected for the short version with 15 items (Sheikh & Yesavage, 1986). Unlike other depression scales, the GDS excluded items of physical and somatic symptoms to provide higher specificity (Balsamo et al., 2018). The scale was validated as a dichotomous response format (yes or no). The total participants included older adults who reported minimal or no symptoms (score of 0–3, 38%), mild level (score of 4–7, 27%), and moderate to severe level (score of 8–15, 35%). The current study did not apply any cutoff in the analyses to take into account the subsyndromal effects (Chuan et al., 2008; Lee et al., 2014).

### 2.4 Informant and clinician rating of depressive symptoms

Neuropsychiatric Inventory (NPI) score was used to assess informant and clinician rating of depressive symptoms. The NPI assesses the presence and severity of multiple neuropsychiatric symptoms of behavior (Choi et al., 2000; Cummings et al., 1994). The instrument is based on the semistructured interview administered to the patients’ informants or caregivers, if available, and ultimately rated by trained clinical psychologists. The three items of affective symptoms, including dysphoria/depression, anxiety, and apathy, were selectively analyzed (Cajanus et al., 2019). The NPI rating scores reflect observable behavioral abnormalities that signify each symptom category. The NPI items were rated as positive if typical features of behaviors are indicated by observation or informant report (NPI‐Dysphoria: acting as if he or she is sad, crying, or spontaneously expressing despair or worthlessness; NPI‐Anxiety: expressing fear, worry, nervousness, or anxiousness when separated with the guardian; NPI‐Apathy: decreased interest and spontaneity in usual activities). Each item was rated from 0 to 3 score across severity levels (0: No symptom, 1: Symptoms causes mild distress, 2: Symptoms are intractable and cause distress, 3: Symptoms are present with major distress).

Though the current dataset contains no available information that can provide reliabilities of informant quality, heterogeneous conditions of informant quality were commonly administered by clinician ratings, which would have minimized the variabilities of informant reports. Also, the severity and caregiver burden were distinctly scored in each NPI item. The administration of NPI finalized by clinicians assured that the severity of each symptom is rated based on the features of reported or observed behaviors, rather than subjective complaints of either patients or informants.

### 2.5 Statistical analysis

Correspondence between the two measurement methods of depression symptoms indicates the convergent validity of the instrument (Campbell & Fiske, 1959; Smith, 2005). The extent of convergence validity of the depression symptom measures was assessed with Kendall's rank‐order correlation (*τ*) between the self‐report scale (GDS) and behavioral rating (NPI‐Dysphoria, NPI‐Anxiety, NPI‐Apathy). The confidence interval of Kendall's tau correlation was calculated with *NSM3* package (95%, 5000 times bootstrap) (Schneider et al., 2020).

We also confirmed whether the differences in the convergence validity are associated with the changes in internal consistency reliability indices. Two reliability coefficients included Cronbach's alpha and the hierarchical coefficient of Omega (McDonald, 2013). Omega hierarchical coefficient is a reliability estimate of the general factor of a set of variables that is based upon the correlation of lower order factors. The omega coefficient above .70 is typically considered an acceptable level. The reliability coefficients were calculated with *psych* package (Revelle, 2018).

The subgrouping within the clinical diagnosis was based on the cutoff quartiles (Q1: below 25th percentile, Q2: 25th or more and below 50th percentile, Q3: 50th or more and below 75th percentile). The CN group was divided into two groups due to the sample size difference (Q1/2: <50th percentile, Q3/4: ≥50th percentile). Convergent validity and reliability analysis were conducted within each subgroup. All of the statistical packages and analyses were conducted under R version 4.2.1.

## 3 RESULT

**FIGURE 1:**
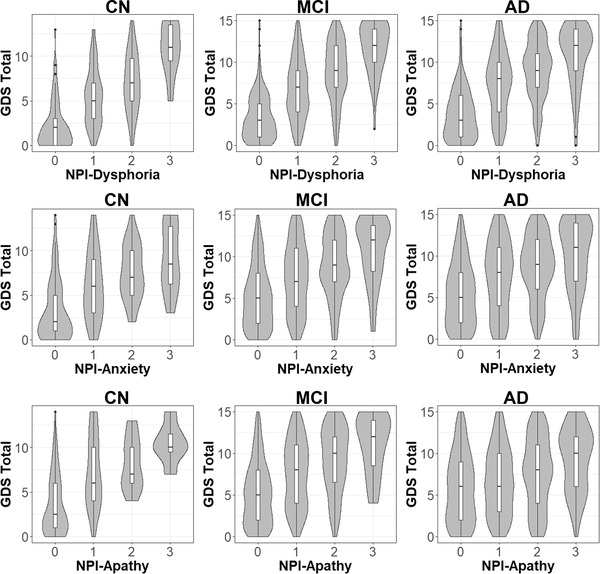
Relationship between self‐report (GDS) and informant/clinician rating (NPI) of depression symptoms by the clinical groups of cognitive impairment. *X*‐axis: NPI item scores indicated by symptom severity ranging from 0 (no symptom) to 3 (major distress). *Y*‐axis: GDS total sum of 15 items. Violin plot indicated relative dispersions of GDS scores in each NPI score. Box plot indicated mean (middle line), 25th to 75th percentile (box) in GDS distributions. CN, cognitively normal; MCI, mild cognitive impairment; AD, Alzheimer's disease dementia; NPI, Neuropsychiatric inventory; GDS, Geriatric Depression Scale.

In the total participants, NPI rating and GDS showed a weak to moderate level of positive correlation in dysphoria (*τ* = .48), anxiety (*τ* = .30), and apathy (*τ* = .25). When the association was examined by the groups of clinical diagnosis (CN, MCI, AD), the corresponding pattern showed that the levels of dispersion in GDS grew larger as a function of the progression of clinical impairment (Figure 1). The correlation showed an apparent decreasing pattern in dysphoria (CN: *τ* = .54, MCI: *τ* = .50, AD: *τ* = .45), anxiety (CN: *τ* = .38, MCI: *τ* = .31, AD: *τ* = .28), and apathy (CN: *τ* = .38, MCI: *τ* = .32, AD: *τ* = .16). The decreasing pattern of correlation was prominent in the correspondence with NPI items of apathy and anxiety.

**FIGURE 2:**
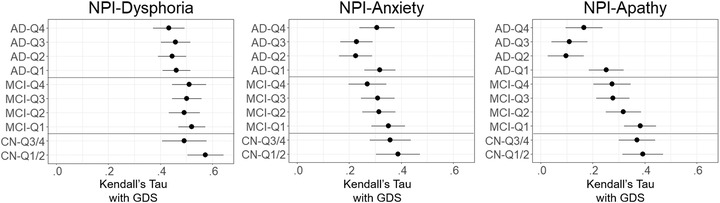
Convergence between self‐report (GDS) and informant/clinician rating (NPI) of depression symptoms across the cognitive function. The strength of convergence is noted with Kendall's tau rank correlation between the two measures (*X*‐axis). Convergence is calculated across clinical groups that were subdivided by quartiles of cognitive function (CERAD‐K) (*Y*‐axis). Error bars indicate 95% confidence interval of Kendall's tau coefficient. CN, cognitively normal; MCI, mild cognitive impairment; AD, Alzheimer's disease dementia; Q1∼4, quartiles within each clinical group from lower to higher cognitive function; GDS, Geriatric Depression Scale.

Diagnoses of cognitive impairment indicated different levels of age and general cognitive functions (Table 1). When subgrouped by cognitive function, the quartiles corresponded with age and education. The cognitive function (CERAD‐K) was correlated with age (*r* = −.47), GDS (*r* = −.18), and NPI (dysphoria: *r* = −.06, anxiety: *r* = −.10, apathy: *r* = −.27; *p*s < .05). The finding showed a consistent pattern when diagnoses of cognitive impairment were subdivided into quartiles (Figure 2). Quartile groups ordered by global cognitive function (CERAD‐K) showed a gradient of decreasing correlation between GDS and NPI, showing that the correspondence generally decreased in the groups of lower cognitive function. In all of the subgroup analyses, confidence intervals (95%) indicated statistically significant (above zero) associations between GDS and NPI scores. The association strength ranged from highest (above average cognitive function in CN, *τ* = .57) to lowest (second quantile cognitive function in AD, *τ* = .22).

Additional reliability analyses were conducted to confirm the possible correspondence between internal consistency and convergent validity (Table 2). In estimating the hierarchical omega coefficient, the four‐factor models (three bifactors and one general factor) were consistent across each subgroup analysis. The overall finding showed that the decreased level of cognitive function was not associated with the gradual deterioration of internal consistency in either of the reliability coefficients. The 95% confidence interval showed a large proportion of overlaps in Cronbach alpha (CN1/2: alpha = .82–.87; MCI‐Q4: alpha = .83–.88; AD‐Q4: alpha = .82–.87).

**TABLE 2:** Internal consistency reliability across clinical diagnosis and quartiles of cognitive function

	Cronbach alpha	Omega‐hierarchical
CN‐Q1/2	.852	.630
CN‐Q3/4	.845	.701
MCI‐Q1	.860	.640
MCI‐Q2	.859	.701
MCI‐Q3	.836	.607
MCI‐Q4	.858	.593
AD‐Q1	.869	.668
AD‐Q2	.868	.641
AD‐Q3	.833	.526
AD‐Q4	.843	.670

Abbreviations: CN, cognitively normal; MCI, mild cognitive impairment; AD, Alzheimer's disease; Q1∼4, quartiles within each clinical group from lower to higher cognitive function.

## 4 DISCUSSION

The current study found that the convergence of depressive symptoms between self‐reported scale (GDS) and informant/clinician rating (NPI) was lower in older adults with cognitive impairment or lower cognitive ability. The deterioration of the convergence between the two measurements corresponded with the lowering levels of cognitive functioning. The disagreements of GDS showed as either false positives (no symptom in NPI but high score in GDS) or false negatives (severe symptom in NPI but low score in GDS). In other words, the presence of cognitive impairment (i.e., MCI and AD dementia) or lower levels of cognitive function is followed by a higher rate of type I and type II errors in using GDS. In postulating the NPI rating as a tenable gold criterion, the convergent validity of GDS may be at risk in the ranges of minimal cognitive impairment.

Previous studies have examined the validity of the self‐report scale of GDS in cognitively impaired older adults, but the studies have suggested inconsistent conclusions about whether GDS remains applicable to the groups of MCI but not in AD dementia (Burke et al., 1989; Conradsson et al., 2013; Lach et al., 2010; Li et al., 2015). Our findings suggest that the loss of convergent validity extends to the subdivided groups of CN and MCI. In the case of dysphoria symptoms, relatively lower cognitive function in CN (Q3 and Q4) was associated with poorer convergence validity comparable to that of the MCI group. In the case of anxiety and apathy symptoms, the convergence validity significantly deteriorated within the MCI group as a function of lower cognitive function. This suggests that even under the same group of clinical diagnosis, the variabilities of cognitive function played role in the maintenance or deterioration of validity. These findings provide further cautious evidence that the validity of self‐reported scales may even be compromised in CN or MCI with lower absolute cognitive functions.

One of the possible factors of the discrepancy between GDS and NPI may be due to how behavioral symptoms of depression manifest. Since the progression of dementia and Alzheimer's pathology affects the overall cognitive capacity that manages affective disturbances, the depressive symptoms in cognitively impaired older adults can be compounded with behavioral dysregulation rather than introspected experiences of discrete emotions (Geda et al., 2013). The NPI reflects a large source of information from the individuals’ overt behavior and can be sensitive to outbursting expressions of emotion. On the contrary, an interoceptive nature of self‐report is more reflective of long‐term personality traits, dysfunctional attitudes, and lower self‐esteem that is not directly observed by observers (Domken et al., 1994; Enns et al., 2000). Self‐appraisals that reflect abstract conceptions of hopelessness and worthlessness may be less associated with emotional outbursts and dysregulation. Other previous literature also suggests that self‐report scales of depression are less responsive to relatively short‐term changes in clinical symptoms contrary to clinician ratings, indicating a dissociating pattern of symptomatology (Sayer et al., 1993).

Anosognosia, or deficit of awareness in self condition, may also underly the currently lowered convergence between the two measurements. Anosognosia has also been observed in the conditions of dementia of AD, and correlational studies have shown that people with AD who are more depressed show more awareness of cognitive impairment (Clare et al., 2012; Mograbi & Morris, 2014). The current study partly supports these previous studies in that the AD dementia group showed mismatching scores of GDS and NPI, which indicates lack of awareness in self‐report of depression symptoms.

However, the remaining side of the mismatched population also needs further discussion. A lowered Kendall's tau may result from both two types of errors: false positive (type 1) or false negative (type 2). The discrepancies are also observed as type 1 errors, which show overly high self‐report scores of GDS while no behavioral observations are supported by NPI rating. Overall, it is assumed that the mismatch between the two measurements occurs from both sides of self‐reporting bias. Unlike anosognosia conditions, cognitively impaired individuals may also easily endorse the GDS items and are unable to reject the familiarity due to more liberal response bias (Deason et al., 2017). Patients with AD show higher positivity bias, more affirming the statements that ask about the presence of depressive symptoms (Bédard et al., 2003).

The current study also examined how subsymptoms of NPI are differentially converged with GDS. The GDS showed relatively stable convergence with dysphoric symptoms, but apathy symptoms markedly diverged with GDS as a function of the severity of the cognitive decline. These results may be primarily due to the difficulty in recollecting and appraising one's daily activities without relying on the current distressful experience. Accurate recollection of past apathy behaviors may have required relatively higher cognitive functions (Do Lam et al., 2012; Fairfield et al., 2015). Moreover, subgroups of lower cognitive function corresponded with higher ages around the late 70s and early 80s that typically accompany multiple physical distress (Hegeman et al., 2015). This frequent physical pain and frailty may be confluent as avolition symptoms without elevating self‐reported depressive symptoms.

Another possible explanation for differing results of NPI items may come from the concurrence of cognitive impairment with behavioral symptoms. Compared with dysphoria, anxiety and apathy symptoms showed a more profound mismatch between the measurements in the dementing population. This pattern may be consistent with the anxiety and apathy (but not dysphoria) symptoms that are more characterized in depressive syndromes under dementia of AD (Brodaty & Connors, 2020). Apathy, for example, has a more evident neural basis in the circuits of the prefrontal and basal ganglia (Levy & Dubois, 2006), and is more reflective of late‐onset depression due to neurodegenerative risks (Kwak et al., 2022). The inability to function in daily life obviously leads to both losses of motivation in the preexisting activities and operant controllability toward surroundings. The additional influx of neurodegenerative changes may only alter behavioral signs that are observed in a similar pattern to signs of the cognitive deficit without mediating self‐reporting mechanisms.

The indication of GDS may deteriorate due to multiple reasons, and the nature of the discrepancy between measures requires further examination. The neuropsychiatric condition may either impede the depth of experiencing current feelings of emotion or integrate beliefs regarding one's past emotional experience (Robinson & Clore, 2002). Pervasive neurodegeneration across widespread cortical regions of the default mode network can disrupt elaborating long‐term emotional experiences (Satpute & Lindquist, 2019).

The current study notes several limitations. While clinicians integrate multiple sources of information on depressive symptoms (informant's report and behavioral observations), the profound variabilities of informant quality can contaminate the validity of the gold criterion. Future studies should acquire whether the ultimate rating of the clinicians was based on acceptable qualities of information. Second, a formal clinical diagnosis of depressive disorder is not confirmed. While separate analyses of three distinctive affective symptoms indicated varying correspondence to the symptomatology, a more tenable diagnostic outcome would clarify the conclusion. Lastly, the current study used a single total score to define the levels of cognitive function. But the effect of cognition on the convergent validity may only hold for specific domains of cognitive function (i.e., episodic memory, executive control, semantic knowledge).

Most importantly, the limitations regarding the source of information require further tasks. Although the formally trained clinicians have applied a uniform criterion that certain behaviors are rated as specific scores, the current study lacks the source and validity of informants. This can lead to systematic bias in rating behavioral symptoms, in that some patients were scored based on a wealth of caregivers’ information, while some other patients lack detailed behavioral history. Moreover, cognitively intact individuals may not have relied less on the informants’ report, which may also bias the decisions made by clinicians.

In conclusion, the results suggest that self‐reported behaviors diverge from observed behaviors of corresponding symptoms in conditions of low cognitive function. Decreased strength of the convergent validity indicates that researchers and clinicians need to be cautious in interpreting the scores of the self‐report scale.

## CONFLICT OF INTEREST STATEMENT

The authors declare no conflicts of interest.

### PEER REVIEW

The peer review history for this article is available at https://publons.com/publon/10.1002/brb3.2898.

## Data Availability

Data available on request due to privacy/ethical restrictions.
